# Cross-Scenario Subdomain Adaptive Displacement Anomaly Detection in Dams

**DOI:** 10.3390/s25102984

**Published:** 2025-05-09

**Authors:** Yu Wang, Guohua Liu

**Affiliations:** College of Civil Engineering and Architecture, Zhejiang University, Hangzhou 310058, China; wangyu97@zju.edu.cn

**Keywords:** dam health monitoring, dam deformation, anomaly detection, transfer learning

## Abstract

To overcome the challenges of limited data, domain distribution differences, and the need for retraining in unsupervised learning methods for cross-scenario anomaly detection in dams, this study introduces a novel approach; the Temporal Displacement Subdomain Adaptation Network (TDSAN) combines temporal convolutional networks with subdomain adaption. This study presents the first application of subdomain adaptation for cross-scenario anomaly detection in dams, addressing distribution shifts across varying operational conditions. The proposed method comprises three key components: a feature extraction network leveraging temporal convolutional layers to capture long-term displacement patterns, a classifier network with fully connected layers to distinguish between normal and anomalous behaviors, and a domain alignment module that uses Local Maximum Mean Discrepancy (LMMD) to align feature distributions between the source and target domains, thereby enhancing the method’s robustness. The approach was validated using data from gravity and arch dams in a specific canyon region in China. The results show that the proposed method demonstrates high classification accuracy and stability in both same-domain and cross-domain scenarios. Compared to other state-of-the-art methods, the proposed approach demonstrates superior classification accuracy and more reliable risk control. This makes it particularly well-suited for cross-domain applications, which are prevalent in real-world engineering scenarios, thereby significantly enhancing its practical applicability.

## 1. Introduction

As a critical infrastructure in hydraulic engineering, the long-term safe operation of concrete dams is directly related to flood control, irrigation, water supply, and ecological security in downstream areas. The safety of dam structures gradually deteriorates due to the combined effects of external loads, temperature fluctuations, material degradation, and inherent defects. To assess the operational status of dams in real time, intelligent monitoring systems are now widely employed. Displacement is a key indicator of dam health [1], providing a direct representation of the dam’s operational condition [2]. However, the measured data often contain missing values or outliers caused by instrument malfunctions or external disturbances. These anomalies may carry critical information that signals potential structural problems. Therefore, investigating the evolution of dam displacement and developing cross-scenario anomaly detection models are essential for intelligent assessment and early warning in dam safety management.

With the advancement of machine learning algorithms, these models have become increasingly prevalent in the field of structural health monitoring for hydraulic engineering due to their robust data processing capabilities [3,4,5,6,7,8,9,10,11]. In anomaly detection, methods are typically categorized into three types based on the availability of labeled data: supervised learning, unsupervised learning, and semi-supervised learning. Supervised learning methods utilize labeled data to train models for anomaly detection, while unsupervised learning methods rely on unlabeled data for the same purpose. Semi-supervised learning approaches combine the strengths of both, initially training models with unlabeled data and subsequently refining them with a small amount of labeled data to enhance the accuracy of anomaly detection [12].

Supervised learning methods detect anomalies by learning the relationship between input variables and labeled categories, constructing classifiers for anomaly detection. In recent years, various supervised learning-based methods have been proposed and validated in practical engineering applications. For instance, Salazar et al. [13] employed an enhanced regression tree model for anomaly detection in dams, while Fisher et al. [14] introduced a method for detecting anomalies in passive seismic data from earth dams. However, their widespread application is limited by the scarcity of labeled data. Unsupervised learning methods, which train models on unlabeled data, have gained popularity in dam monitoring. Ji et al. [15] used the DBSCAN clustering algorithm to detect anomalies in concrete dams, and Lei et al. [16] applied clustering analysis for deformation early warning. Despite their effectiveness, these methods often struggle with multi-factor coupling features, which involve complex, non-linear, and dynamic relationships among variables. Semi-supervised learning methods combine the advantages of both supervised and unsupervised approaches by pre-training on unlabeled data and fine-tuning with limited labeled data. Su et al. [17] and Dong et al. [18] applied semi-supervised learning to improve anomaly detection in dam structural behavior and tailings dam monitoring, respectively. However, these methods still rely heavily on the accuracy of model construction [19] and face challenges in addressing multi-factor coupling features, which refer to the intricate interactions and dependencies among multiple variables, often exhibiting non-linear and dynamic relationships.

**Figure 1:**
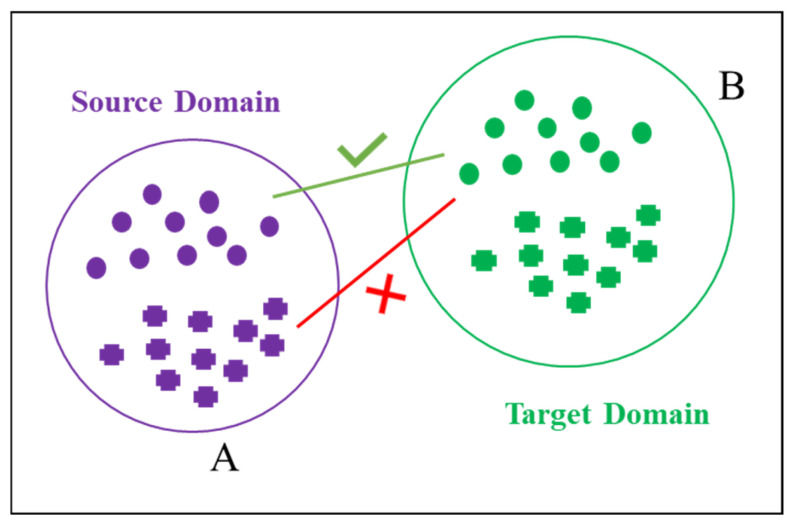
The learning-based domain adaptation method. (**A**) represents the source domain, shown in purple; (**B**) represents the target domain, shown in green.

The continuous advancement of transfer learning techniques has significantly contributed to the growing popularity of transfer learning-based methods in structural health monitoring. Transfer learning improves task performance in the target domain by utilizing knowledge from the source domain, making it especially useful when target domain data is scarce or labeled data is limited, as shown in Figure 1. Transfer learning can be broadly categorized into traditional transfer learning and domain-adaptive transfer learning, depending on the application. These methods have been widely applied in areas such as bridge health monitoring [20,21,22,23], machinery fault diagnosis [24,25,26], and wind turbine health monitoring [27,28,29].

Traditional transfer learning methods, which transfer knowledge from the source domain to the target domain, have been widely applied in structural health monitoring. For example, Wang et al. [30] developed an efficient deep learning model using transfer learning to accurately predict the expansion of concrete cracks in bridges under complex environmental conditions. Deng et al. [31] integrated drone-based image acquisition with three-dimensional scene reconstruction to develop a transfer learning-driven model for detecting pier cracks. Okazaki et al. [32] employed a region-based convolutional neural network combined with transfer learning to detect concrete cracks in complex backgrounds, significantly improving the detection accuracy through efficient feature extraction and offering a novel solution for structural damage monitoring in challenging environments. Li et al. [33] proposed a model that leverages multiple deep learning and transfer learning techniques to facilitate knowledge transfer within dams, enabling the assessment of structural responses. Despite these advancements, traditional transfer learning methods often encounter limitations due to substantial discrepancies in data distribution between the source and target domains, making distribution mismatch a major obstacle to practical implementation.

Domain-adaptive transfer learning mitigates distribution discrepancies between the source and target domains by aligning features and facilitating cross-domain knowledge transfer, thereby improving model performance. This approach has been widely explored in structural health monitoring within civil engineering. For example, Liao et al. [34] proposed a Discriminative Wavelet Adversarial Adaptation Network to assess the health status of similar structures using diagnostic information from reference structures. Ren et al. [35] applied Variational Mode Decomposition and Multiscale Permutation Entropy for feature extraction, integrating these methods with transfer learning to develop a wind turbine health assessment model, which demonstrated high effectiveness across diverse environmental conditions. Lin et al. [36] introduced a cross-scenario damage localization method based on a Deep Adaptive Network, which optimizes feature extraction to identify damage-sensitive and domain-invariant features, significantly enhancing the accuracy of damage detection in simply supported beams.

Transfer learning is still in the nascent stage of application within the field of dam health monitoring. Current research mainly focuses on crack damage detection and anomaly identification within single-dam scenarios, while systematic methods and theoretical frameworks for cross-scenario prediction and anomaly detection remain underdeveloped. Existing methods for dam anomaly detection face three key challenges: (1) limited data and scarce labels in the target domain, which hinder effective model training; (2) significant distribution differences between the source and target domains, affecting the transferability of knowledge; and (3) the need for retraining and fine-tuning in unsupervised and self-supervised learning methods when applied across scenarios, which limits their ability to meet real-time and dynamic monitoring needs.

To address the aforementioned issues, this paper presents TDSAN, a novel cross-scenario displacement anomaly detection method based on temporal convolutional networks and subdomain adaptation, which is applied for the first time in the field of cross-scenario dam anomaly detection. TDSAN utilizes subdomain adaptation to effectively learn from the source domain and adapt to the target domain, which overcomes the issue of limited labels in the target domain, thereby enhancing model training and overall performance. Additionally, TDSAN employs Local Maximum Mean Discrepancy (LMMD) to align the subdomain feature distributions between the source and target domains, minimizing distribution shifts across different scenarios. This alignment ensures that the model maintains a robust detection performance in cross-scenario applications. Moreover, TDSAN’s ability to generalize across scenarios without requiring extensive retraining makes it particularly efficient in meeting the real-time and dynamic monitoring needs of practical applications, such as dam anomaly detection.

The structure of the paper is organized as follows: Section 2 details the implementation of the TDSAN method. Section 3 outlines the case study analysis process. Section 4 presents a comparison between the proposed method and widely used existing methods, including a discussion of the results. Finally, Section 5 concludes the paper and proposes directions for future research.

## 2. Methodology

**Figure 2:**
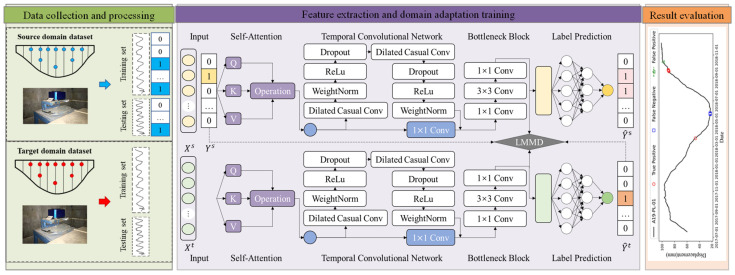
Cross-Scenario Displacement Anomaly Detection Method for Dams Based on the TDSAN Framework.

This study presents a method for cross-scenario dam anomaly detection using the TDSAN framework. Figure 2 illustrates the overall framework, which consists of three phases: dataset creation, feature extraction with domain adaptation training, and result evaluation. In the first phase, data from both the source and target domains are automatically collected through the dam monitoring system. These data are preprocessed, including normalization, and anomaly labels are manually assigned to the source domain based on expert knowledge. The features and labels are then split into training and testing sets according to a specified ratio. In the second phase, a self-attention mechanism [37] filters the extracted features, while a temporal convolutional network is used to extract features from both the source and target domain data. A Bottleneck structure aligns the features, followed by training using Local Maximum Mean Discrepancy. Finally, in the third phase, a classifier network detects anomalies to identify potential issues in the target domain. The results of the anomaly detection are then evaluated, and the model’s performance is assessed using various evaluation metrics. The following section provides the implementation details of the proposed method.

### 2.1. Construction of Multi-Scenario Dam Displacement Anomaly Detection Dataset

The proposed method requires processing horizontal data from multiple dam scenarios to construct source and target domain datasets, as shown in Figure 2. For dam displacement detection, “multiple dam scenarios” refers to the detection scenarios for different parts of a single dam or the detection scenarios for different dams. The construction process primarily involves two aspects: data acquisition and data processing. In terms of data acquisition, it is essential to clearly identify the sources of data for both the source and target domains. Source domain data are typically selected from dams with high monitoring quality, complete data, and stable displacement changes, while target domain data are obtained from the dams to be analyzed for anomaly detection, with no specific requirements regarding dam type or data quality. The required source and target domain data are then collected through sensors installed in the dam structures. Regarding data processing, the source and target domain data are processed separately, with steps such as data cleaning, normalization, time-series transformation, and label generation performed independently. Additionally, the transformations applied during data preprocessing must remain consistent to ensure comparability and consistency across data from different dams.

It is important to note that the source domain dataset includes both features and labels. The features consist of the dam’s horizontal data, while the labels indicate whether the data are anomalous (1 for anomaly, 0 for normal). These labels can be manually assigned based on historical experience and expert knowledge or generated using automated labeling methods based on statistical features. The anomalies discussed in this paper are outliers that deviate from the expected evolution of the sequence for various reasons, without further categorization. In contrast, the target domain dataset only contains displacement features without labeled anomaly states, making anomaly detection in the target domain an unsupervised learning process. This approach to constructing the source and target domain datasets introduces data distribution differences between them, effectively simulating the variability in data characteristics across different scenarios in real-world engineering applications.

### 2.2. TDSAN Framework Construction

**Figure 3:**
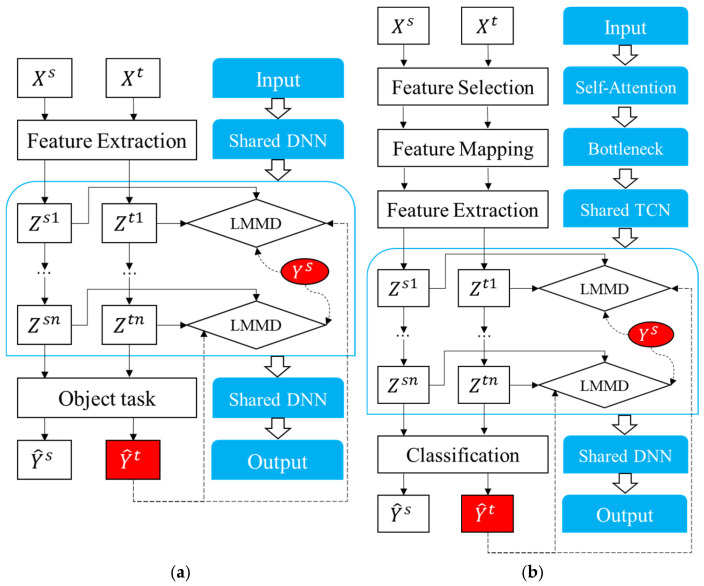
DSAN and TDSAN Frameworks. (**a**) DSAN. (**b**) TDSAN.

The TDSAN method proposed in this section is an improvement upon the DSAN [38] method. Figure 3a illustrates the details of the DSAN network, which is composed of three main components: feature extraction, LMMD training module, and target detection. First, the feature extraction module processes the data from both the source and target domains using a shared convolutional neural network to extract deep feature representations. This module maps the input features from the source domain $X_{s}$ and the target domain $X_{t}$ into the same feature space, providing a foundation for subsequent feature alignment and classification. Next, the LMMD training module aligns the features from both domains across multiple layers of feature representations, reducing the distributional differences between them. By aligning the feature distributions at each layer, the LMMD module ensures that the target domain data are processed more effectively within the framework trained on the source domain, thereby improving the similarity between the source and target domains. Finally, the target detection module classifies the samples using the aligned features through a classifier, generating classification results $Y_{s}$ for the source domain and pseudo-labels $Y_{t}$ for the target domain.

**Figure 4:**
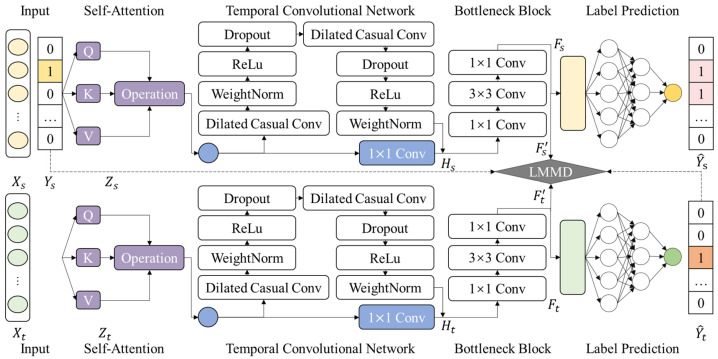
Implementation Details of TDSAN.

This section presents an anomaly detection model designed to address the discrepancies in feature distributions between the source and target domains. The model improves upon the traditional DSAN approach by integrating feature selection and transformation modules, thereby enhancing performance in cross-scenario anomaly detection tasks. Figure 3b illustrates the construction process of TDSAN, while Figure 4 provides a detailed implementation of the network. The model comprises five key components: feature selection, feature extraction, feature transformation, domain alignment, and target detection.

The feature selection module employs a self-attention mechanism, generating Queries, Keys, and Values to calculate their correlations and produce attention weights. The feature transformation module utilizes a Bottleneck structure with three convolutional layers: the first layer reduces dimensions using 1 × 1 convolution, the second layer extracts features in the compressed space using 3 × 3 convolutions, and the third layer adjusts feature dimensions with 1 × 1 convolution. The feature extraction module is based on a temporal convolutional network that employs dilated convolutions to expand the receptive field and capture long-term dependencies, while causal convolutions ensure temporal consistency, making it well-suited for time series data. Finally, the target detection module consists of a classifier network with three fully connected layers: the first and second layer extract the input features, and the third layer generates classification labels through Softmax, enabling the final prediction of samples from both the source and target domains.

As shown in the figure, given the source domain input $X_{s}$ and the target domain input $X_{t}$, the data first pass through a shared self-attention mechanism layer to obtain the source domain feature representation $Z_{s}$ and the target domain feature representation $Z_{t}$. Next, $Z_{s}$ and $Z_{t}$ undergo feature extraction using TCN, resulting in $H_{s}$ and $H_{t}$, which captures the temporal dependency features in the input data. The features are then processed by the feature transformation module, where the source and target domain features are compressed and transformed into $F_{s}$ and $F_{t}$, respectively, to reduce redundancy and extract key features. Subsequently, these features are input into the LMMD module for alignment, where the distribution differences between the source and target domain features are minimized, ensuring that they are consistently distributed in the shared latent space, resulting in aligned features $F_{s}^{'}$ and $F_{t}^{'}$. These aligned features are then passed through the classifier network to generate the source domain classification labels ${\hat{Y}}_{s}$ and target domain pseudo-labels ${\hat{Y}}_{t}$. The classification results ${\hat{Y}}_{s}$ for the source domain features are compared with their true labels $Y_{s}$, and the model is optimized by minimizing the supervised loss. For the target domain features, which lack true labels, only pseudo-labels ${\hat{Y}}_{t}$ are generated, and unsupervised alignment and pseudo-supervised training are performed using these pseudo-labels.

### 2.3. Implementation Procedures of TDSAN

The training process of TDSAN is outlined in Algorithm 1. Given the source domain samples $X_{s}$ along with their corresponding labels $Y_{s}$, and the target domain samples $X_{t}$, the data undergo feature selection, extraction, and transformation to produce $F_{s}$ and $F_{t}$. These features are then passed through the classifier to generate the predicted source domain labels ${\hat{Y}}_{s}$ and target domain pseudo-labels ${\hat{Y}}_{t}$. The objective of the source domain classification loss *i + s* is to minimize the error between the predicted labels ${\hat{Y}}_{s}$ and the true labels $Y_{s}$, which is computed using the cross-entropy loss as follows:

(1) $$L_{\mathrm{src}\text{\_}\mathrm{cls}}=\mathrm{CrossEntropy}\left(\hat{Y_{S}},Y_{S}\right)$$

The objective of the domain alignment loss is to minimize the distribution discrepancy between the source and target domains. First, a Gaussian kernel is used to estimate the similarity between the source and target domain features, which is calculated as follows:

(2) $$k\left(x_{i},x_{j}\right)=\exp\left(-\frac{|x_{i}-x_{j}{|}^{2}}{2\sigma^{2}}\right)$$

(3) $$K\left(F_{S},F_{T}\right)=\sum_{i=1}^{M}\exp\left(-\frac{|F_{S}-F_{T}{|}^{2}}{2\sigma_{i}^{2}}\right)$$

where $K\left(F_{S},F_{T}\right)$ represents the multi-scale Gaussian kernel, which is the result of stacking Gaussian kernels with different bandwidths.

Next, the weight matrix for aligning the subdomains is calculated using the class labels to compute the feature weights for different categories in both the source and target domains as follows:

(4) $$Y_{s}^{o}=OneHot\left(y_{s}\right)$$

(5) $$Y_{t}^{o}=Softmax\left({\hat{Y}}_{t}\right)$$

(6) $$w_{s}=\frac{Y_{s}^{o}}{\sum Y_{s}^{o}}$$

(7) $$w_{t}=\frac{Y_{t}^{o}}{\sum Y_{t}^{o}}$$

(8) $$W_{ss}=w_{s}w_{s}^{T}$$

(9) $$W_{tt}=w_{t}w_{t}^{T}$$

(10) $$W_{st}=w_{s}w_{t}^{T}$$

where $Y_{s}^{o}$ and $Y_{t}^{o}$ represent the encoded labels for the source and target domains, respectively; $w_{s}$ and $w_{t}$ are the weights for the source and target domains; and $W_{ss}$, $W_{tt}$, and $W_{st}$ are the weighted matrices for the source domain, target domain, and between the source and target domains, respectively. By combining the weights and the kernel function for both the source and target domains, the final LMMD loss can be expressed as follows:

(11) $$L_{LMMD}=Tr\left(W_{ss}K_{ss}\right)+Tr\left(W_{tt}K_{tt}\right)-2Tr\left(W_{st}K_{st}\right)$$

where $K_{ss}$, $K_{tt}$, and $K_{st}$ represent the kernel matrices for the source domain and source domain, target domain and target domain, and source domain and target domain, respectively; $Tr\left(\cdot \right)$ denotes the trace operation of a matrix.

By combining the source domain classification loss and the LMMD alignment loss, the overall loss is computed as follows:

(12) $$L_{total}=\lambda_{1}L_{\mathrm{src}\text{\_}\mathrm{cls}}+\lambda_{2}L_{LMMD}$$

Finally, the total loss $L_{total}$ is used for backpropagation to compute the gradients of the model parameters, and gradient descent is applied to update the model parameters as follows:

(13) $$\theta =\theta -\eta \nabla_{\theta }L_{\mathrm{total}}$$

**Algorithm 1:** TDSAN training processInput: source domain sample $X_{s}$, source label $Y_{s}$, target domain sample $X_{t}$, hyperparameters $\lambda_{1}$, $\lambda_{2}$ 1.  Randomly initialize model parameters 2.  for all i=1, 2, …, n do: 3.     //Feature screening, extraction, and transformation 4.     $F_{si}=Bottleneck\left(TCN\left(\mathrm{Self}\text{\_}\mathrm{attention}\left(X_{si}\right)\right)\right)$ 5.     $F_{ti}=Bottleneck\left(TCN\left(\mathrm{Self}\text{\_}\mathrm{attention}\left(X_{ti}\right)\right)\right)$ 6.     //Classification 7.     ${\hat{Y}}_{si}=\mathrm{Classifier}\left(F_{si}\right)$ 8.     ${\hat{Y}}_{ti}=\mathrm{Classifier}\left(F_{ti}\right)$ 9.     //Calculate loss 10.   Get source classification loss $L_{\mathrm{src}\text{\_}\mathrm{cls}}$ by $Y_{si}$ and ${\hat{Y}}_{si}$ 11.   Get LMMD loss $L_{LMMD}$ by $F_{ti}$, $F_{si}$, $Y_{si}$ and ${\hat{Y}}_{ti}$ 12.   Get total loss $L_{total}$ by $L_{\mathrm{src}\text{\_}\mathrm{cls}}$ and $L_{LMMD}$ 13.   //Backpropagation and parameter update 14.   Update model by $L_{total}$ 15. end Output:${\hat{Y}}_{t}$


### 2.4. Evaluation Metrics

This study evaluates the performance of the model using five metrics: accuracy, $F_{1}$, Area Under the Receiver Operating Characteristic Curve (AUROC) [39], Source Risk (SR) [40], and Target Risk (TR) [41]. Among these, accuracy and precision primarily assess the overall classification performance of the model, while the latter three further evaluate the model’s classification performance on the source and target domains.

Accuracy represents the proportion of correctly detected positive and negative classes, and is calculated as follows:

(14) $$\mathrm{Acc}=\frac{\mathrm{TP}+\mathrm{TN}}{\mathrm{all}}$$

Precision represents the proportion of true positive samples among all the samples predicted as positive by the model, and is calculated as follows:

(15) $$\mathrm{precision}=\frac{\mathrm{TP}}{\mathrm{TP}+\mathrm{FP}}$$

(16) $$\mathrm{recall}=\frac{\mathrm{TP}}{\mathrm{TP}+\mathrm{FN}}$$

(17) $$F_{1}=\frac{2\times precision\times recall}{precision+recall}$$

where TP represents the number of correctly identified anomalous points, FP represents the number of points incorrectly classified as anomalous, and all denotes the total amount of data.

AUROC is used to measure the model’s discriminatory ability in classification tasks. Specifically, it calculates the area under the ROC curve at different thresholds, reflecting the model’s overall performance in distinguishing between positive and negative samples. The formula is as follows:

(18) $$\mathrm{AUROC}=\int_{0}^{1}\mathrm{TPR}\left(\mathrm{FPR}\right)\;d\left(\mathrm{FPR}\right)$$

where $\mathrm{TPR}=\frac{\mathrm{TP}}{\mathrm{TP}+\mathrm{FN}}$ and $\mathrm{FPR}=\frac{\mathrm{FP}}{\mathrm{FP}+\mathrm{TN}}$, TP represents the number of correctly identified anomalous points, FP represents the number of points incorrectly classified as anomalous, and FN represents the number of anomalous points that were missed.

SR is used to assess the model’s performance on source domain data. It is calculated by evaluating the model’s error using the cross-entropy loss on the source domain test set. Specifically, it computes the cross-entropy loss between the source domain data and the true labels, using expectation. The formula is as follows:

(19) $$R_{\mathrm{SR}}\left(m\right)=E_{x_{s}∼P_{s}}\left[\mathrm{ce}\left(m\left(x_{s}\right),y_{s}\right)\right]$$

TR is used to evaluate the model’s performance on target domain data. Its calculation method is similar to that of Source Risk. The formula is as follows:

(20) $$R_{\mathrm{TR}}=E_{x_{t}∼P_{t}}\left[\mathrm{ce}\left(m\left(x_{t}\right),y_{t}\right)\right]$$

All the metrics mentioned above, except for SR, require labeled data from the target domain. In this section, target domain labels were manually assigned to validate the effectiveness of the method. However, in practical applications, this method does not necessarily require labels from the target domain.

## 3. Case Study

This section constructs a cross-scenario dam anomaly detection dataset using data obtained from three real-world dam projects, which is used to validate the proposed TDSAN method. This dataset will also be used in the next section for comparative analysis and discussion.

### 3.1. Project Overview

**Figure 5:**
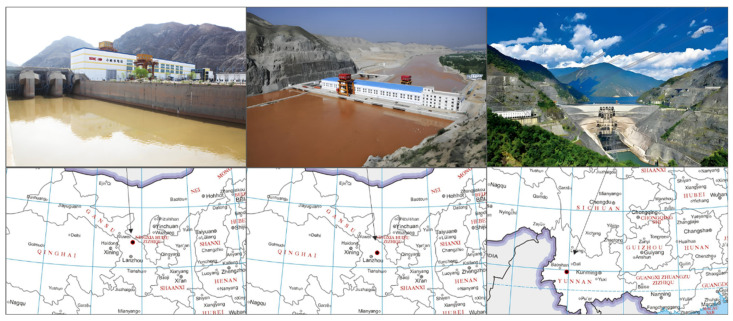
Locations and Outlines of the Selected Dams.

This section evaluates the A, B, and C dam projects as case studies for analysis. Figure 5 shows the locations and outlines of the three dams.

Dam A is located along the main channel of the Yellow River in Lanzhou City, Gansu Province. It is a power station in the river channel, designed as a concrete gravity dam with a top elevation of 1502.00 m, a height of 50.7 m, and a total reservoir capacity of 48 million m^3^. The main structure was completed in September 2004, the reservoir began storing water, and the first generator set started operating on 8 November 2004.

Dam B is located in Jingyuan County, Gansu Province. The dam has a total length of 297 m at the top, with a top elevation of 1438.50 m and a height of 55 m. The reservoir has a total capacity of 23.68 million m^3^. Construction officially began on 15 November 2005, and the first generator set was connected to the grid and began power generation on 31 October 2008.

Dam C is located in the middle reaches of the Lancang River in Yunnan Province. The reservoir’s checked flood level is 1242.51 m, with a total capacity of 15 billion m^3^, and a maximum height of 294.5 m. The normal water level is 1240.00 m, the dead water level is 1166.00 m, and the regulation capacity is 9.9 billion m^3^. Construction officially began in January 2002, and on 16 December 2008, the reservoir began to store water.

### 3.2. Dataset

**Figure 6:**
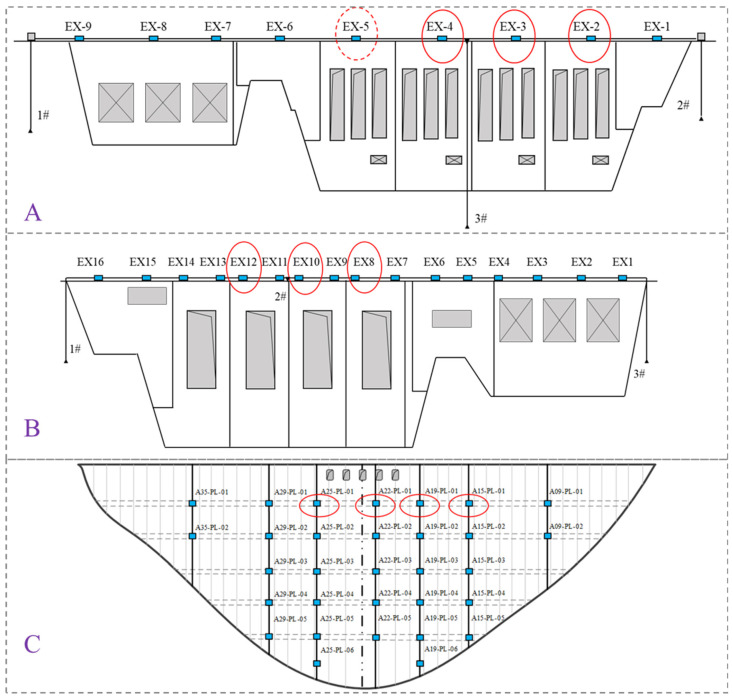
Horizontal Displacement Monitoring Layout for Each Dam.

The horizontal data for dams A, B, and C were collected through sensors installed within the dam structures. Figure 6 illustrates the layout of the displacement monitoring sensor networks for each dam.

As shown in the figure, the horizontal displacement at the top of dam A is monitored using a combination of plumb line and taut wire methods. A total of 9 measurement points are set along the taut wire, numbered EX-1 to EX-9, with an inverted plumb line at each end. The taut wire is measured using the NARI RY-20S capacitive taut wire instrument, with a data collection frequency of once per day, while the inverted plumb lines are measured using the NARI RZ-50S capacitive bidirectional plumb line coordinate instrument, also with a collection frequency of once per day.

At dam B, an inverted plumb line is arranged at each end of the dam crest, numbered IP1 and IP3, to calibrate the endpoints of the taut wire. The plumb lines are monitored automatically using the NARI RZ-25 capacitive bidirectional plumb line coordinate instrument, with a monitoring frequency of once per day. The horizontal displacement at the dam crest is observed using the taut wire method. A taut wire is installed at a height of 0 + 003.30 m on the dam crest, with a total length of 293.50 m, and 16 measurement points, numbered EX1 to EX16. The taut wire is monitored automatically using the NARI RY-20 capacitive taut wire instrument, with a monitoring frequency of once per day.

At dam C, vertical plumb lines are set in the corridors of nine dam sections (4#, 9#, 15#, 19#, 22#, 25#, 29#, 35#, and 41#) at elevations of 1014 m, 1054 m, 1100 m, 1150 m, and 1190 m. In sections 4#, 9#, 35#, and 41#, the vertical plumb lines extend into the grouting corridors on both sides of the dam, allowing for monitoring of the deformation of the dam foundation on both sides. These plumb lines are connected within the foundation corridors, grouting corridors, and inverted plumb lines, to monitor the horizontal deformation and deflection of the dam body. The plumb lines are monitored automatically using the NARI capacitive plumb line coordinate instrument, with a monitoring frequency of three times per day.

Specifically, the horizontal displacement monitoring data from measurement point EX-5 on dam A is selected as the source domain data, denoted as “0”, and is highlighted in the figure with a red dashed circle. Additionally, data from 10 other measurement points, marked with solid red circles in the figure, are selected from dams A, B, and C as the target domain data. Based on the source domain data from EX-5, three-stage transfer is performed, progressively moving from nearby to distant measurement points on the same dam and on dams B and C, as detailed in Table 1. In scenario 1, data from measurement points EX-4, EX-3, and EX-2 on dam A are selected for transfer detection, denoted as “1”, “2”, and “3”, respectively. In scenario 2, data from measurement points EX-12, EX-10, and EX-8 on dam B are selected for transfer detection, denoted as “4”, “5”, and “6”, respectively. In scenario 3, data from measurement points A25-PL-01, A22-PL-01, A19-PL-01, and A15-PL-01 on dam C are selected for transfer detection, denoted as “7”, “8”, “9”, and “10”, respectively.

For the selection of measurement points, dam A and dam B use measurement points at the dam crest, while dam C uses measurement points at an elevation of 1190 m. There are three reasons for this choice: first, measurement points at higher elevations experience larger displacements, which facilitates comparison and annotation; second, these measurement points have relatively complete sequential data, which contributes to analysis results; and third, the selected points represent different spatial ranges and dam types, allowing for a comprehensive assessment of the method’s robustness and cross-scenario generalization ability.

**Figure 7:**
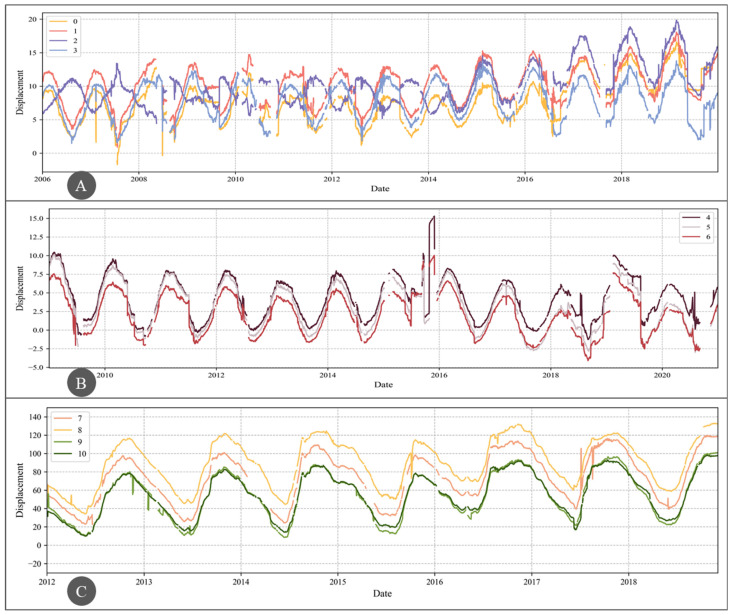
Horizontal Displacement Evolution at Measurement Points in the Three Dams.

Figure 7 illustrates the long-term evolution of horizontal displacement at the selected measurement points. The sample time range for the four measurement points on dam A is from 1 January 2006 to 31 December 2019; for the three measurement points on dam B, it is from 1 January 2009 to 31 December 2020; and for the four measurement points on dam C, it is from 1 January 2012 to 3 December 2018. The sampling frequency for all data is once per day. As can be seen, the data length for dam A is longer than that for dam B, and the data length for dam B is longer than that for dam C, which is consistent with the dataset construction principles described earlier. The sequences in the figure contain missing data, outliers, and other anomalies. To facilitate the subsequent results evaluation, the data were labeled using expert knowledge and historical experience, with normal values labeled as 0 and anomalous values labeled as 1.

### 3.3. Network Setup

All models in this study were executed in an environment with Python 3.7 and PyTorch 1.7. The computer configuration includes an Intel(R) Core(TM) i7-8700K CPU @ 3.70 GHz and an NVIDIA GeForce RTX 2080 Ti with 11 GB of memory (Intel Corporation, Santa Clara, CA, USA; NVIDIA Corporation, Santa Clara, CA, USA).

To facilitate model training, this study packages the source domain data features and their corresponding labels, as well as the feature data from each target domain, and splits them into training and testing sets in a 7:3 ratio. To optimize the model’s performance across diverse tasks, hyperparameter tuning is conducted using a grid search method. Hyperparameters are selected via uniform sampling from predefined value ranges, and through multiple iterations the optimal parameter combination is identified. The tuning results are presented in Table 2. For model optimization, the Adam optimizer is used, with a weight decay parameter set to 0.0001, β1 set to 0.5, and β2 set to 0.99.

### 3.4. Training Results

The hyperparameters have been set in the previous section. In this section, the TDSAN method is trained based on the hyperparameter design results. During the training process, the source domain loss, LMMD loss, and total loss for each iteration are recorded.

**Figure 8:**
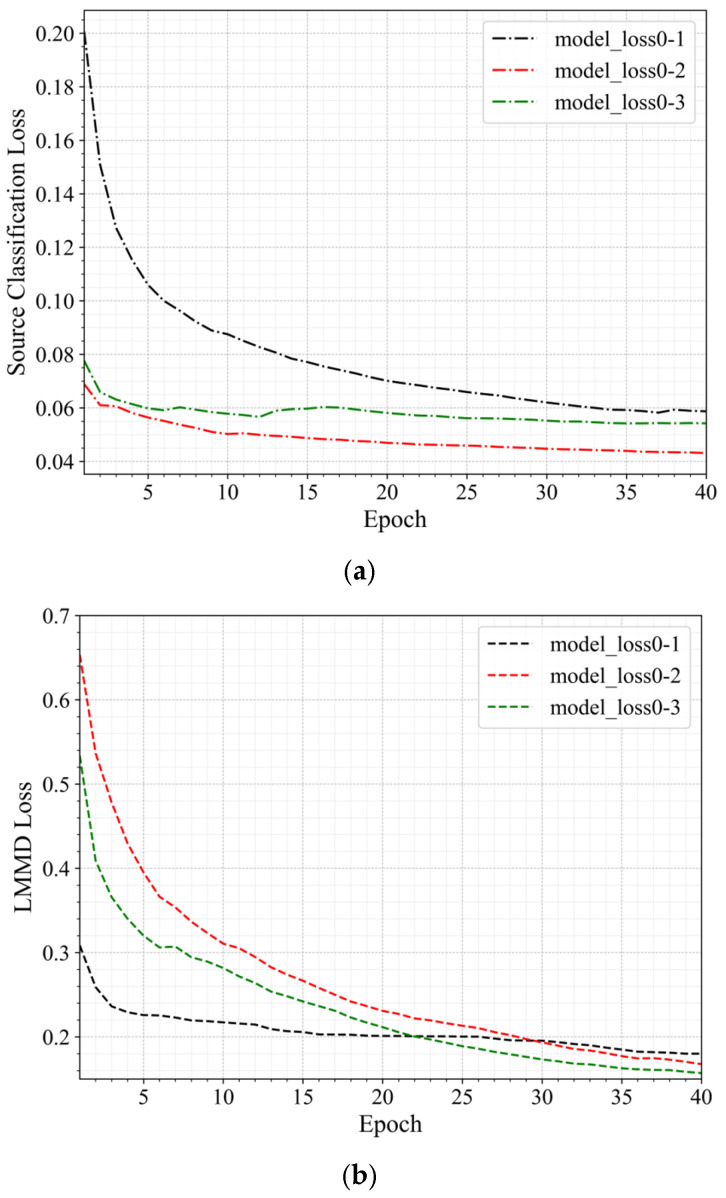

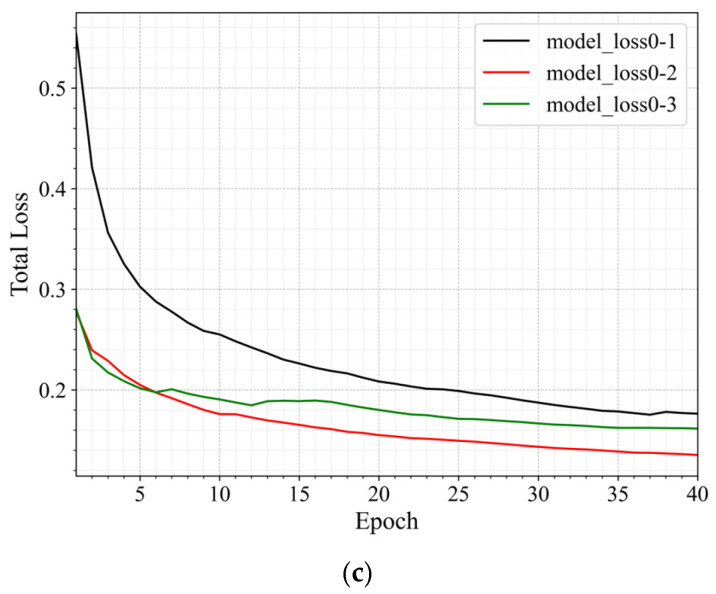
Loss Curves of Same-Domain Transfer Scenario. (**a**) The Source Domain Loss Curve. (**b**) The LMMD Loss Curve. (**c**) The Total Loss Curve.

Figure 8 shows the changes in the various loss values during the training process for the same-domain transfer scenarios 0–1, 0–2, and 0–3. In the figure, different colors represent different transfer scenarios: black for scenario 0–1, red for scenario 0–2, and green for scenario 0–3. Different line styles represent different types of loss: dotted lines for source domain loss, dashed lines for LMMD loss, and solid lines for total loss. As training progresses, both the source domain loss and the total loss gradually decrease, indicating an improvement in the model’s classification performance on the source domain and that training is approaching convergence. While the LMMD loss fluctuates, it shows an overall decreasing trend, suggesting that the distributional differences between the source and target domains are gradually narrowing.

**Figure 9:**
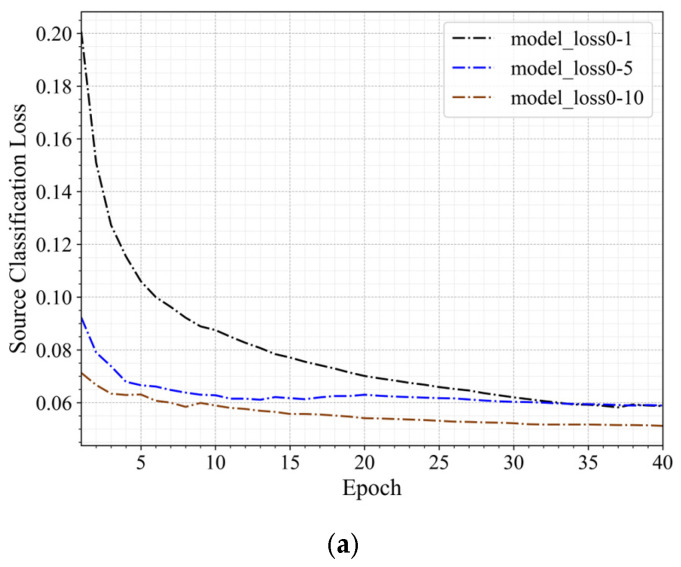

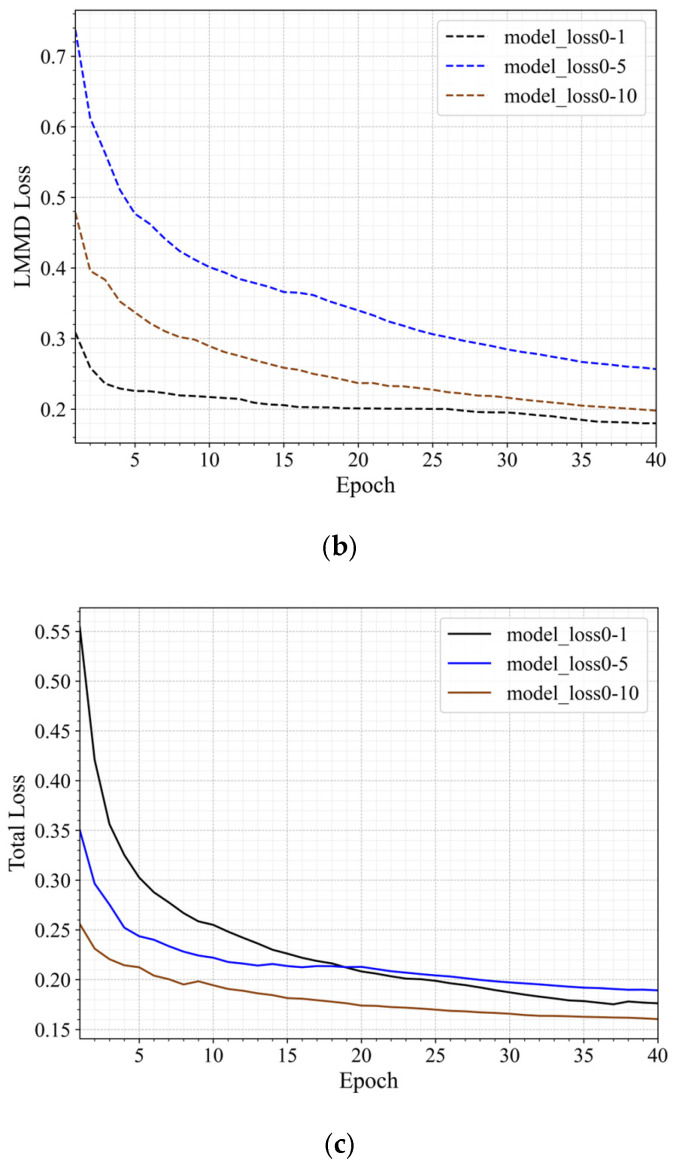
Loss Curves of Three Transfer Scenarios with Varying Distances. (**a**) The Source Domain Loss Curve. (**b**) The LMMD Loss Curve. (**c**) The Total Loss Curve.

Figure 9 shows the changes in the loss values during the training process for the three transfer scenarios 0–1, 0–5, and 0–10, arranged from near to far. As in Figure 6, different colors represent different transfer scenarios: blue for scenario 0–5 and brown for scenario 0–10. The figure shows that the loss values exhibit different trends depending on the migration scenario. As the distance between the transfer scenarios increases, the rate of decrease in the loss values slows down, particularly in scenario 0–10, where the reduction in total loss and LMMD loss is relatively small. This indicates that as the difference between the target and source domains grows, the model’s convergence speed slows. In all scenarios, the source domain loss gradually decreases, indicating that the model is adapting to the source domain data. Meanwhile, the LMMD loss and total loss demonstrate the model’s adaptation process to the distributional differences between the source and target domains across different transfer scenarios.

### 3.5. Results Analysis

**Figure 10:**
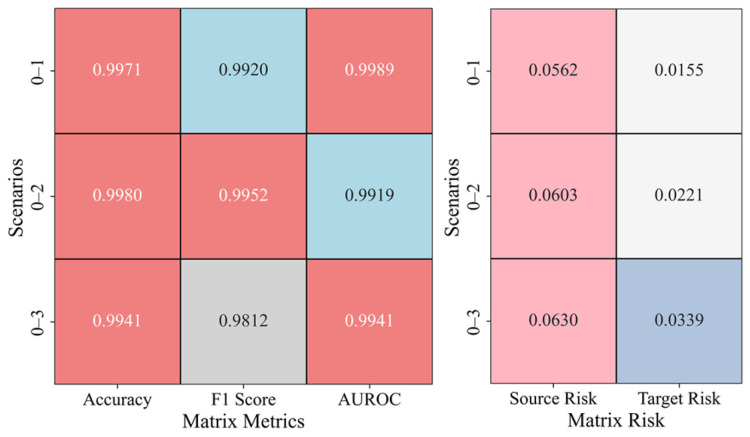
Anomaly Detection Results in Same-Domain Transfer Scenario.

Based on the settings described above, the proposed method was utilized for anomaly detection in various transfer scenarios. The analysis and discussion considers different cases, including same-domain transfer, cross-domain transfer, and transfer scenarios with varying distances. The results of the above scenarios were analyzed using the evaluation metrics and risk metrics proposed earlier in the text. The evaluation metrics include accuracy, F1 score, and AUROC, while the risk metrics include source risk and target risk. The results are as follows:

In the same-domain transfer scenarios, Figure 10 shows the anomaly detection evaluation matrix and the risk matrix for scenarios 0–1, 0–2, and 0–3. For the evaluation matrix, higher metric values indicate a stronger classification performance by the model. For the risk matrix, smaller metric values suggest that the model’s fitting error between the source and target domains is lower. In the evaluation matrix, accuracy and AUROC are close to 1, indicating high classification accuracy; however, the F1 score slightly decreases in scenario 0–3, suggesting that as the complexity of the scenario increases, the balance between positive and negative sample classification weakens. The risk matrix shows that as the complexity of the scenario increases, the source risk gradually rises; however, the target risk remains consistently lower than the source risk, indicating that the transfer learning model effectively mitigates risk in the target domain.

**Figure 11:**
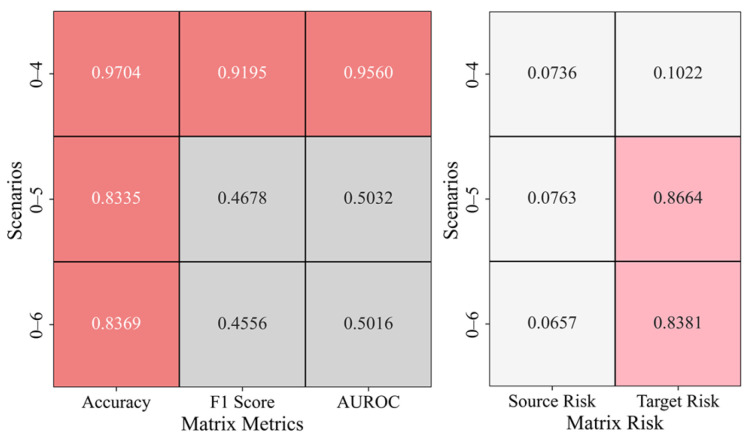
Anomaly Detection Results in the A–B Cross-Domain Transfer Scenario.

In the cross-domain transfer scenarios, Figure 11 presents the results of transfer learning from gravity dam A to gravity dam B, including the anomaly detection evaluation matrix and risk matrix for scenarios 0–4, 0–5, and 0–6. In the evaluation matrix, accuracy is relatively high in scenario 0–4, but in the transition from scenario 0–4 to scenario 0–6, both the accuracy and F1 score fluctuate, indicating that the model’s performance is unstable in scenarios with varying complexity. Notably, in scenarios 0–5 and 0–6, AUROC decreases significantly, limiting the model’s ability to distinguish between positive and negative samples. In the risk matrix, the source risk varies inconsistently across different scenarios, suggesting that data distribution or feature differences affect the source domain’s adaptability. The target risk increases significantly in scenarios 0–5 and 0–6, indicating that the transfer learning model’s risk transfer capability diminishes in more complex scenarios.

**Figure 12:**
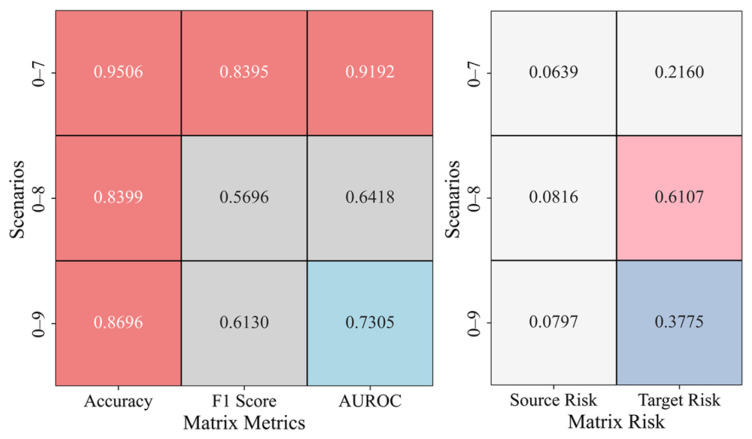
Anomaly Detection Results in the A–C Cross-Domain Transfer Scenario.

Figure 12 presents the results of cross-domain transfer learning from gravity dam A to arch dam C in different river basins, including the evaluation and risk matrices for scenarios 0–7, 0–8, and 0–9. In the evaluation matrix, scenario 0–7 performs well, with an accuracy of 0.9506, an F1 score of 0.8395, and an AUROC of 0.9192, demonstrating strong classification performance. However, in scenarios 0–8 and 0–9, both the F1 score and AUROC decrease, indicating that the model’s performance fluctuates when faced with more complex scenarios. Nonetheless, some recovery is observed in scenario 0–9, suggesting that the model exhibits adaptability. In the risk matrix, both the source and target risks in scenario 0–7 are relatively low, indicating that transfer learning has effectively controlled risk in the target domain. In contrast, in scenarios 0–8 and 0–9, the target risk increases significantly, suggesting a decline in the model’s ability to mitigate risk in more complex scenarios. However, in scenario 0–9, the target risk decreases, potentially due to changes in the target domain data distribution or improved model adaptability.

**Figure 13:**
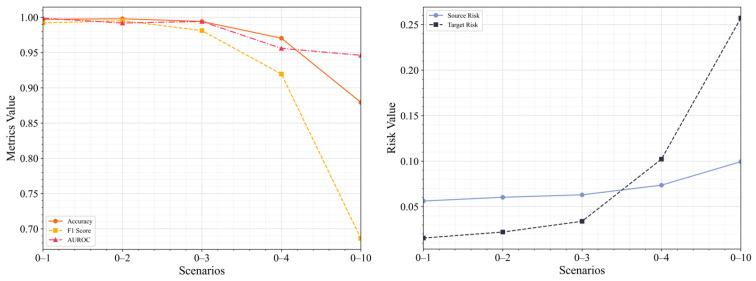
Anomaly Detection Results in Transfer Scenarios with Varying Distances.

In the progressively distant transfer scenarios, Figure 13 presents the anomaly detection evaluation matrix and the risk matrix for five scenarios: 0–1, 0–2, 0–3, 0–4, and 0–10. As the transfer distance increases, significant changes in model performance are observed. In terms of evaluation metrics, accuracy remains close to 1 in the 0–1 and 0–2 scenarios, indicating strong performance in short-range same-domain migrations. However, in long-distance cross-domain scenarios (e.g., 0–4 and 0–10), accuracy declines, dropping to 0.8794 in scenario 0–10, suggesting a degradation in classification performance. Both the F1 score and AUROC reflect similar trends, with precision and discriminatory ability decreasing in long-distance scenarios, although AUROC remains relatively high in scenario 0–10, indicating that the model retains some discriminatory power.

In terms of risk analysis, the source risk increases slightly with migration distance, rising from 0.0562 in scenario 0–1 to 0.0994 in scenario 0–10, indicating a minor increase in source domain fitting error. Target risk, however, rises significantly in cross-domain transfer scenarios, particularly in scenario 0–10, where it increases to 0.2572 from 0.0155 in scenario 0–1, highlighting the model’s diminished risk control capability in the target domain as the migration distance grows.

Overall, the model demonstrates a stable classification performance in same-domain transfer tasks, with good fitting between the source and target domains. In cross-domain transfer tasks, the model still performs well in classification, but its ability to balance positive and negative samples and maintain consistent classification performance becomes unstable. Source risk remains consistently below 0.1, while target risk increases significantly with the complexity of the scenario. In progressively distant transfer tasks, as the migration distance increases, the model’s anomaly detection capability decreases, and the risk metrics show an upward trend.

## 4. Comparison and Discussion

To comprehensively evaluate the performance of the TDSAN method, this section compares its anomaly detection results with those of the DSAN [38], DANN [42], and MMDA [43] methods. Since TDSAN is an improvement upon DSAN, comparing it with DSAN helps to validate the necessity of the proposed modifications. DANN, a widely used method in cross-scenario tasks, is compared with TDSAN to highlight its superiority. The MMDA method, which performs well in handling multimodal data and cross-scenario applications, is included in the comparison to further demonstrate the effectiveness of TDSAN. By comparing these methods, the detection performance and practical effectiveness of TDSAN are thoroughly demonstrated.

### 4.1. Experimental Setup

Before training each model, hyperparameter optimization is performed using a search method to ensure that each model achieves optimal performance for the target task. Similarly, the dataset is divided into training and testing sets in a 7:3 ratio. During model tuning, the Adam optimizer is used, with the weight decay parameter set to 0.0001, β1 set to 0.5, and β2 set to 0.99.

To ensure consistency across all methods, the batch size is set to 32, the number of epochs to 40, and the timestep to 10, values that remain consistent with those used in the TDSAN method. In the DSAN model, the feature extraction layer uses a convolutional neural network, with the kernel size as a tunable hyperparameter. The results of hyperparameter optimization are shown in Table 3.

In the DANN model, in addition to the learning rate, there are two other hyperparameters: the source domain loss weight and the domain adaptation loss weight. The results of hyperparameter tuning are shown in Table 4.

In the MMDA model, in addition to the learning rate, there are four other hyperparameters: the source domain classification loss weight, MMD loss weight, target alignment loss weight, and distribution discrepancy loss weight. The results of hyperparameter tuning are shown in Table 5.

### 4.2. Model Comparison and Discussion

To comprehensively compare and analyze these models, this section will focus on evaluating each model’s anomaly detection metrics and risk assessment metrics across different scenarios. The analysis is divided into two main parts: same-domain transfer scenario comparison and cross-domain transfer scenario comparison.

#### 4.2.1. Same-Domain Transfer Scenario

**Figure 14:**
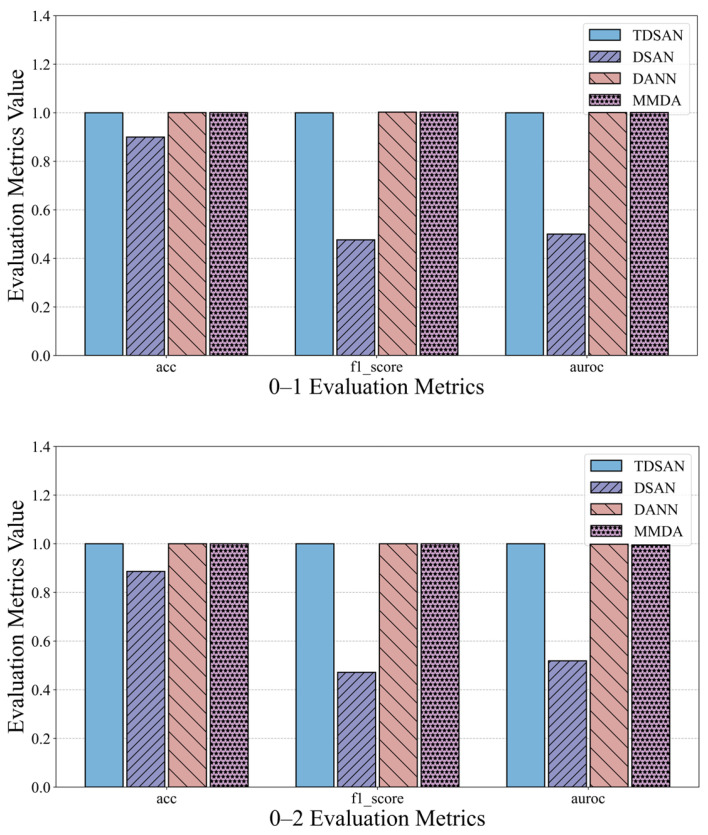

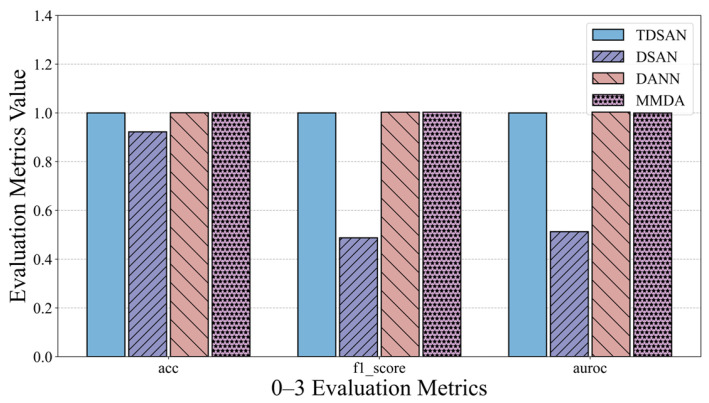
Comparison of Detection and Evaluation Results in Same-Domain Transfer Scenario.

The anomaly detection results for each method in the same-domain transfer scenario are compared in Figure 14, which presents the evaluation metrics for scenarios 0–1, 0–2, and 0–3. To simplify the analysis, the results for the TDSAN method are normalized to a baseline value of 1, with the evaluation values of the other methods expressed as ratios relative to this baseline. For key metrics such as accuracy, precision, and AUC, a higher ratio indicates better classification performance, while a lower ratio suggests poorer performance. In terms of accuracy, the DSAN method falls below the baseline, indicating inferior classification performance compared to TDSAN. Conversely, the DANN and MMDA methods achieve accuracy values close to or exceeding the baseline, demonstrating strong overall performance. Regarding precision, the DSAN method shows a ratio significantly below 1, approaching 0.5 in several scenarios, which reflects inadequate classification performance. In contrast, the DANN and MMDA methods consistently maintain ratios close to or slightly above 1, indicating better precision and stability. For the AUC metric, the DSAN method underperforms relative to TDSAN across all scenarios, with the most significant gap observed in scenario 0–2. Meanwhile, the AUC values for the DANN and MMDA methods are generally close to or surpass the baseline, highlighting their superior classification capabilities.

In summary, the DSAN method performs worse than TDSAN in terms of accuracy, precision, and AUC, validating the necessity of the improvements made to the DSAN method. The DANN and MMDA methods show results that are close to or slightly higher than TDSAN on some metrics, but they are somewhat less stable and consistent overall, indicating that TDSAN demonstrates superior performance in the same-domain transfer scenario.

**Figure 15:**
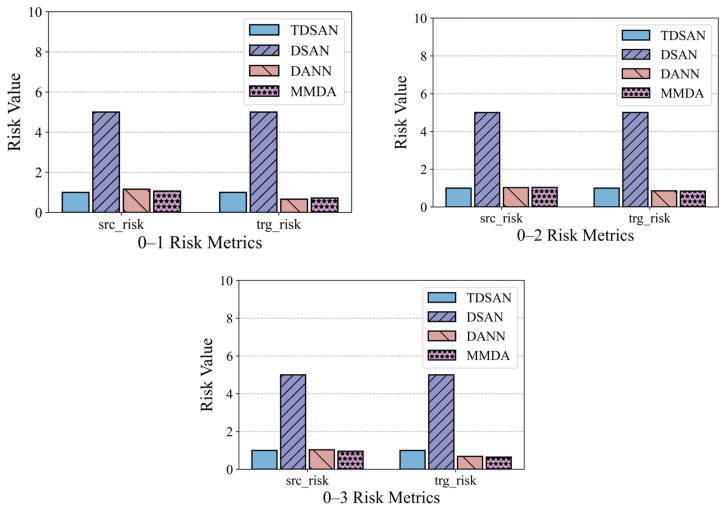
Comparison of Risk Assessment Results in Same Domain.

Figure 15 illustrates the risk assessment metrics for the same-domain transfer scenarios. To facilitate comparison, the results for the TDSAN method are normalized to a baseline value of 1, with any ratio exceeding 5 capped at 5. The vertical axis represents the ratio of the evaluation values for other methods relative to the TDSAN baseline. A lower ratio indicates that the classification fitting error between the source and target domains is smaller relative to TDSAN, suggesting better performance. The results reveal that the DSAN method exhibits significantly higher ratios for both source and target domain risk metrics compared to TDSAN, particularly in scenarios 0–1, 0–2, and 0–3, where the source domain risk ratio reaches 5. This highlights notable deficiencies in DSAN’s ability to control classification errors in both domains. In contrast, the DANN and MMDA methods show source domain risk ratios that are either close to or slightly higher than TDSAN in most scenarios, indicating that their ability to control classification fitting errors in source domain transfer scenarios is comparable to that of TDSAN.

In summary, the TDSAN method outperforms the DSAN method in terms of risk control for both the source and target domains. Furthermore, it demonstrates comparable performance to the DANN and MMDA methods in most scenarios, highlighting its advantage in controlling classification errors.

#### 4.2.2. Cross-Domain Transfer Scenario

**Figure 16:**
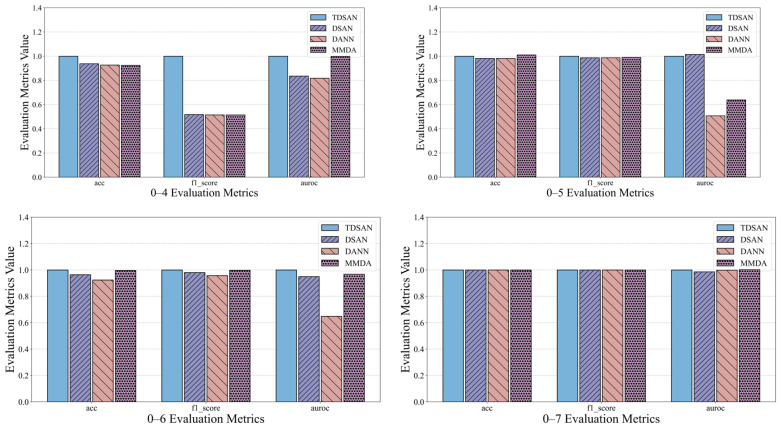

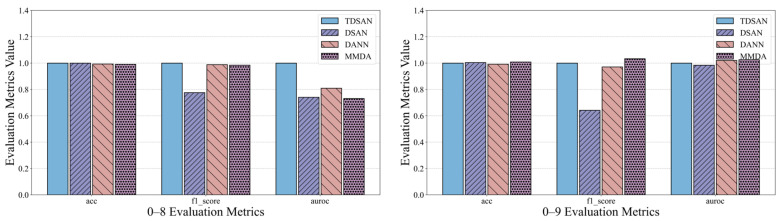
Comparison of Anomaly Detection Results in A–C Cross-Domain Transfer Scenario.

Figure 16 presents the performance of TDSAN, DSAN, DANN, and MMDA across three evaluation criteria: accuracy, precision, and AUC, within the cross-domain transfer scenarios A–B and A–C. In terms of accuracy, both DSAN and DANN consistently perform worse than TDSAN across all scenarios, while MMDA shows a performance similar to or slightly better than TDSAN in some cases. Regarding precision, the F1 scores for all three methods are lower than those for TDSAN, with fluctuations across different scenarios indicating that their ability to balance positive and negative samples is less effective than TDSAN. For the AUC criterion, both DSAN and DANN fall significantly below TDSAN in all scenarios, with DANN exhibiting notable fluctuations in some instances. The MMDA method’s AUC is close to or slightly exceeds TDSAN in certain scenarios, but it shows greater overall variability, reflecting weaker stability.

Overall, TDSAN outperforms the other methods in classification accuracy, balancing positive and negative samples, and distinguishing between samples, demonstrating exceptional robustness. This success is largely due to key improvements in TDSAN, including the use of the LMMD loss function, which effectively aligns cross-domain distributions and mitigates the instability issues observed with DANN, as well as optimized strategies for feature extraction and alignment. In contrast, while DANN and MMDA perform well in certain scenarios, they exhibit slightly lower overall stability. The DSAN method performs poorly across all metrics, suggesting that it is unsuitable for transfer tasks involving significant differences between the source and target domains.

**Figure 17:**
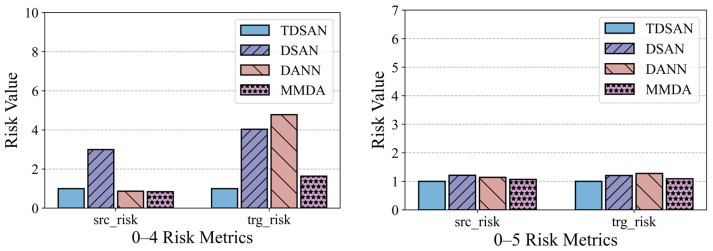

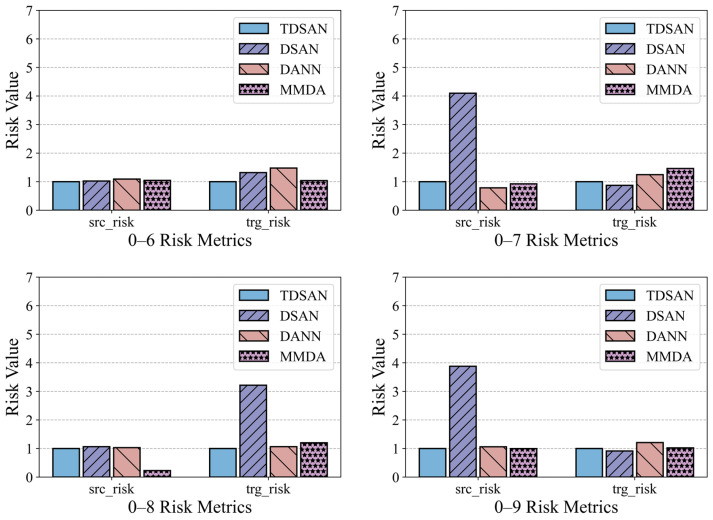
Comparison of Risk Assessment Results in A–B and A–C Cross-Domain Transfer Scenarios.

Figure 17 presents the risk assessment results for cross-domain transfer from gravity dam A to both the same-basin arch dam B and the different-basin arch dam C. In terms of the source domain risk, the DSAN method exhibits significantly higher risk values than TDSAN in several scenarios, particularly in 0–4, 0–7, and 0–9, indicating greater classification errors in the source domain. The DANN and MMDA methods show source domain risks close to TDSAN, with MMDA performing slightly better than TDSAN in scenario 0–4. Regarding the target domain risk, DSAN also displays significantly higher risk values than TDSAN in most scenarios, especially in scenario 0–8, reflecting limited classification ability in the target domain. DANN’s target domain risk is similar to TDSAN, though slightly higher in scenario 0–6, highlighting its limitations in target domain classification. MMDA’s performance in the target domain varies, showing slightly better results than TDSAN in scenario 0–7, but higher risk values in scenario 0–8. Overall, TDSAN outperforms the other methods in risk assessment for both the source and target domains, demonstrating its superior applicability and generalization ability in cross-domain transfer tasks.

## 5. Conclusions

This study introduces TDSAN, a cross-scenario dam displacement anomaly detection method that leverages TCN and subdomain adaptation. The method utilizes TCN for feature extraction and incorporates LMMD to capture relevant information from the known domain. Through domain alignment, this information is transferred to the target domain, facilitating cross-scenario anomaly detection. This research also offers a detailed analysis of how to construct a multi-scenario dam displacement anomaly detection dataset and evaluates the method’s performance across various domain modes. The key findings of the study are summarized as follows:

1. TDSAN performs exceptionally well in same-domain transfer tasks, demonstrating strong classification ability and the capacity to effectively distinguish between positive and negative samples. Additionally, it aligns the source and target domains efficiently. In cross-domain transfer tasks, despite the influence of scenario fluctuations, the model sustains high classification accuracy and stabilizes the balance between positive and negative samples while reducing the target domain risk.
2. An analysis of the method’s performance across different transfer distances shows that as the transfer distance increases, the model’s anomaly detection ability decreases, while the risk metrics exhibit an increasing trend.
3. The proposed method is compared with three baseline models using real engineering case studies. In the same-domain transfer tasks, the results indicate that TDSAN performs similarly to DANN and MMDA in some aspects, while surpassing DSAN. In the cross-domain transfer tasks, TDSAN exhibits superior adaptability, with enhanced classification performance and more effective risk control compared to the other methods.

The method effectively adapts to anomaly pattern transfer across multiple dams and scenarios, with classification capabilities that meet practical engineering requirements. It demonstrates a strong generalization ability and can provide technical support for intelligent dam monitoring and management systems.

## Figures and Tables

**Table 1:** Considered Transfer Scenarios.

Scenario 1	Scenario 2	Scenario 3
0→1	0→4	0→7
0→2	0→5	0→8
0→3	0→6	0→9
		0→10

**Table 2:** Hyperparameters for TDSAN.

Hyperparameter	Optimum Range	Optimum Results
Batch size	[32, 64]	32
Epoch	200	40
Learning rate	[1 × 10^−2^, 1 × 10^−3^, 1 × 10^−4^]	0.0001
Timestep	[5, 10, 30]	10
TCN kernel size	[8, 16, 32]	16
$\lambda_{1}$	[0.1:10]	1.87
$\lambda_{2}$	[0.1:10]	2.08

**Table 3:** Hyperparameters of DSAN.

Hyperparameter	Optimum Range	Optimum Results
Learning rate	[1 × 10^−2^, 1 × 10^−3^, 1 × 10^−4^]	0.0001
CNN kernel size	[8, 16, 32]	32
$\lambda_{1}$	[0.1:10]	1.87
$\lambda_{2}$	[0.1:10]	1.59

**Table 4:** Hyperparameters of DANN.

Hyperparameter	Optimum Range	Optimum Results
Learning rate	[1 × 10^−2^, 1 × 10^−3^, 1 × 10^−4^]	0.001
$w_{s}$	[0.1:10]	5.14
$w_{d}$	[0.1:10]	2.94

**Table 5:** Hyperparameters of MMDA.

Hyperparameter	Optimum Range	Optimum Results
Learning rate	[1 × 10^−2^, 1 × 10^−3^, 1 × 10^−4^]	0.001
$w_{1}$	[0.1:10]	1.38
$w_{2}$	[0.1:10]	8.37
$w_{3}$	[0.1:10]	3.96
$w_{4}$	[0.1:10]	6.79

## Data Availability

Data utilized in this work are available from the corresponding author by request.
